# Use of “Surface Analyzer” to evaluate the Effect of Two Polishing Systems on Surface Texture of Four Newer Composites

**DOI:** 10.5005/jp-journals-10005-1524

**Published:** 2018-08-01

**Authors:** Shefally Garg, Munish Goel, Shweta Verma, Nanika Mahajan, Bhawna Kaul, Vikas Garg

**Affiliations:** 1 Senior Lecturer, Department of Conservative Dentistry and Endodontics Himachal Dental College, Sundernagar, Himachal Pradesh, India; 2 Professor and Head, Department of Conservative Dentistry and Endodontics Himachal Dental College, Sundernagar, Himachal Pradesh, India; 3 Reader, Department of Conservative Dentistry and Endodontics Himachal Dental College, Sundernagar, Himachal Pradesh, India; 4 Registrar, Department of Pedodontics, Indira Gandhi Government Dental College & Hospital, Jammu, India; 5 Registrar, Department of Pedodontics, Indira Gandhi Government Dental College & Hospital, Jammu, India; 6 Senior Lecturer, Department of Orthodontics and Dentofacial Orthopedics Surendera Dental College & Research Institute, Sri Ganganagar Rajasthan, India

**Keywords:** Composites, Light cure unit, Mylar strip, Polishing system, Profilometer.

## Abstract

Composites polymerized with a clear matrix on the surface will leave a resin-rich surface layer that is easily abraded in the oral environment, exposing unpolished, rough, inorganic filler material. The purpose of this study was to evaluate the polishing effect of two different polishing systems: One-step (PoGo) system and Sof-Lex (multistep) system on four different resin composites: Synergy D6, Clearfil APX Esthetics, Filtek Z 350 XT, Ceram X Mono. After polishing, the specimens were analyzed for average surface roughness using a two-dimensional surface profilometer.

**How to cite this article:** Garg S, Goel M, Verma S, Mahajan N, Kaul B, Garg V. Use of “Surface Analyzer” to evaluate the Effect of Two Polishing Systems on Surface Texture of Four Newer Composites. Int J Clin Pediatr Dent 2018;11(4):266-270.

## INTRODUCTION

Composites are direct restorative materials composed of three components that include resin matrix, filler particles, and silane coupling agent.^1^ The smooth surface can be achieved by polymerizing composite resin with a Mylar strip. However, diamond and carbide burs are necessary for the anatomic contour of the surface of teeth.

This procedure can induce porosities or cracks on the tooth-restoration interface.^2^ Instruments used for finishing and polishing tooth-colored restorative materials include carbide finishing burs, 25 to 50 m diamond finishing burs, abrasive-coated rubber cups and points, aluminum oxide-coated abrasive discs, strips, and polishing pastes.^3^

Polishing is performed after finishing that removes scratches from the surface of a restoration to produce a smooth, light-reflective luster.^4^ The ultimate esthetics and shade of these tooth colored restoratives are strongly influenced by the final surface polish.^5^ Smooth restorations can be easily maintained compared with restorations with rougher surfaces.

Polishing reduces plaque retention, gingival irritation, staining, and recurrent caries.^6^ Finishing systems are used in a dry field with a slow-speed bur and a light, intermittent pressure. Before polishing, the finished surface has its final contour and it should be defect-free.

## MATERIALS AND METHODS

**Figs 1A and B:**
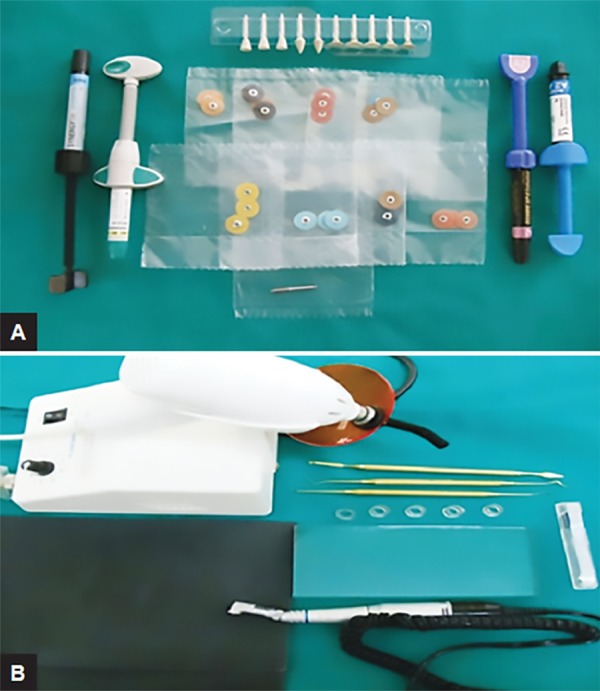
(A) Composites with PoGo and Sof-Lex polishing systems. (B) Dent America Light Cure unit and Teflon composite manipulating instruments

The resin composites used in this study were (Fig. 1A): (1) Ceram X Mono (DENTSPLY, York, Pennsylvania, USA), (2) Filtek Z 350 XT (3M ESPE, St. Paul, Minnesota, USA), (3) Clearfil APX Esthetics (KURARAY, Okayama, Japan), and (4) Synergy D6 (COLTENE WHALEDENT, Ohio, USA). A total of 60 specimens (15 specimens of each of the restorative material) were fabricated in cylindrical rubber base molds 8 mm in diameter and 10 mm in height by polymerizing successive 2 mm thick layer for 30 seconds with light activating source which provides a luminous intensity of 450 mw/cm^2^ at a wavelength of 480 nm (Dent America, USA) (Fig. 1B). On the base of the mold, a glass slab was placed. The resin composites were placed using a Teflon-coated plastic instrument.

The mold was slightly overfilled with a composite resin and a Mylar strip was placed on the top side of the mold. A 2 mm thick glass slide was placed on the strip for surface flattening and to extrude the excess material. The curing of samples was done for 40 seconds through the Mylar strip and glass slide.

**Figs 2A to E:**
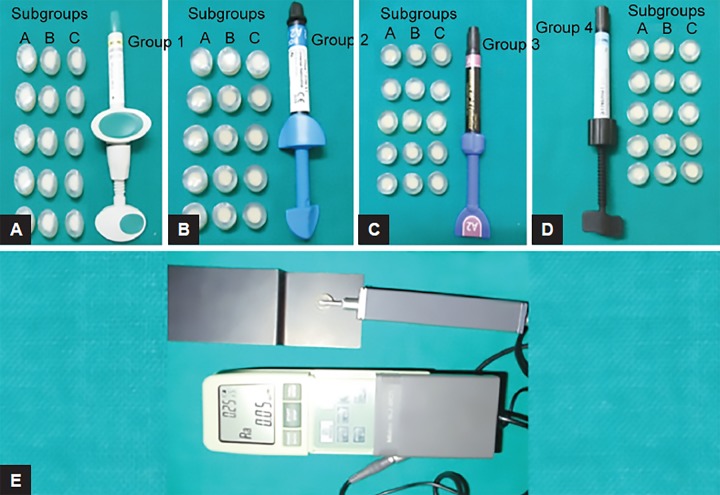
(A) Fifteen samples prepared with Ceram X Mono, (B) fifteen samples prepared with Filtek Z 350 XT, (C) fifteen samples prepared with Clearfil APX Esthetics, (D) fifteen samples prepared with Synergy D6, and (E) sample reading on profilometer

An additional 20 seconds curing was done on both sides of the specimens after removing the Mylar strip and a glass slab. Samples were divided into four groups of 15 samples each of all four light-cure composites as follows (Figs 2A to D): group I:Ceram X Mono group; group II: Filtek Z 350 XT; group III: Clearfil APX Esthetics; and group IV: Synergy D6. Each group is subdivided into three subgroups of five samples each as follows: subgroup A: cured with a Mylar strip and were not polished; subgroup B: polished with PoGo polishing system; and subgroup C: polished with Sof-Lex polishing system.

Ten samples of each subgroups B and C were wetly grounded with a silicon carbide paper to provide a baseline before using the polishing systems. The specimens were then polished by the same operator using the PoGo system and Sof-Lex system containing polishing discs and paste (Fig. 1A), as per manufacturer’s instructions; 20 samples were polished with a Sof-Lex system (3M ESPE, St. Paul, Minnesota, USA).

These are color coded aluminum oxide discs, from darker shades (coarser grits) to lighter shades (fine grits). Disks were attached by a metal hub to the autoclavable metal mandrel. The coarse grit disks was used for surface reduction at a speed of 10,000 rpm with light pressure for 15 seconds and then rinsed and dried with air-water syringe for 6 seconds.

The medium grit disks were used for contouring at a speed of 10,000 rpm for 15 to 20 seconds with light pressure for 15 seconds and then samples were rinsed and dried with air-water syringe for 6 seconds. The fine grit disk followed by superfine grit disk was used to finish at high speed of 30,000 rpm for 15 to 20 seconds each with light pressure for 15 seconds and then rinsed and dried with air-water syringe for 6 seconds.

Rest of the 20 samples were polished with PoGo (DENTSPLY, York, Pennsylvania, USA) system at a speed of 20,000 rpm. These are gray discs used in the dry field for polishing with light pressure for 15 seconds and then rinsed and dried with air-water syringe for 6 seconds. Both the polishing systems were used according to the manufacturer’s instructions.

After the specimens were polished, they were analyzed for surface roughness using a two-dimensional surface mechanical profilometer (Mitutoyo Surftest SJ-201, Japan) (Fig. 2E). A profilometer is a device with a diamond stylus of 2 diameter to trace a fixed linear distance on the surface of the samples. It produces a digital tracing and it calculates the average surface roughness (Ra) value.

The Ra of a specimen was defined as the arithmetic average height of roughness component; irregularities from the mean line were measured within the sampling length. The profilometer readings were made at the center of each specimen. This provides a quantitative recording of surface irregularities. After an evaluation of Ra, results were subjected to statistical analysis by using one-way analysis of variance (ANOVA) test.

## RESULTS

Table 1 shows the mean values for Ra (μm) of four composite resin materials when finished with PoGo finishing and polishing system. The mean surface roughness was highest for Clearfil APX Esthetics (1.21 ± 0.19), followed by Synergy D6 (1.02 ± 0.21), Filtek Z 350 XT (0.79 ± 0.05), and Ceram X Mono (0.64 ± 0.05). This difference found was statistically significant (p < 0.001).

Table 2 shows the mean values for Ra (μm) of four composite resin materials when finished with Sof-Lex finishing and polishing System. Mean surface roughness was highest for Synergy D6 (1.24 ± 0.13) followed by Clearfil APX Esthetics (1.03 ± 0.08), Filtek Z 350 XT (0.52 ± 0.05), and Ceram X Mono (0.34 ± 0.04) when finished with Sof-Lex polishing system. This difference found was statistically significant (p < 0.001).

Table 3 shows the mean values for Ra (μm) of four composite resin materials when finished with Mylar strip. Mean surface roughness was highest for Ceram X Mono (0.56 ± 0.05), followed by Filtek Z 350 XT (0.14 ± 0.03), Clearfil APX Esthetics (0.06 ± 0.05), and Synergy D6 (0.06 ± 0.04) when finished with Mylar strip polishing system. This difference was statistically significant (p < 0.001).

**Table 1:** **Table 1:** Mean values for average surface roughness (Ra) (μm) of four composite resin materials when finished with Pogo finishing and polishing system

								*95% confidence interval*							
*Composite materials*		*n*		*Mean*		*SD*		*Lower limit*		*Upper limit*		*f-value*		*p-value*	
Ceram X Mono		5		0.64		0.05		0.56		0.7		13.75		<0.001**	
Filtek Z 350 XT		5		0.79		0.05		0.72		0.85					
Clearfil APX Esthetics		5		1.21		0.198		0.97		1.45					
Synergy D6		5		1.02		0.21		0.72		1.25					

** p < 0.001 is highly significant

**Table 2:** **Table 2:** Mean values for average surface roughness (Ra) (μm) of four composite resin materials when finished with Sof-Lex finishing and polishing system

								*95% confidence interval*							
*Composite materials*		*n*		*Mean*		*SD*		*Lower limit*		*Upper limit*		*f-value*		*p-value*	
Ceram X Mono		5		0.34		0.04		0.30		0.39		84.31		<0.001**	
Filtek Z 350 XT		5		0.52		0.05		0.46		0.58					
Clearfil APX Esthetics		5		1.03		0.08		0.92		1.14					
Synergy D6		5		1.24		0.13		0.07		0.40					

** p < 0.001 is highly significant

**Table 3:** **Table 3:** Mean values for average surface roughness (Ra) (μm) of four composite resin materials when finished with Mylar strip

								*95% confidence interval*							
*Composite materials*		*n*		*Mean*		*SD*		*Lower limit*		*Upper limit*		*f-value*		*p-value*	
Ceram X Mono		5		0.56		0.05		0.50		0.62		154.53		<0.001**	
Filtek Z 350 XT		5		0.14		0.03		0.09		0.17					
Clearfil APX Esthetics		5		0.06		0.05		0.003		0.12					
Synergy D6		5		0.06		0.04		0.02		0.12					

** p < 0.001 is highly significant

## DISCUSSION

A composite restoration cannot be differentiated from the surrounding enamel surface. So, polished restorations should exhibit an enamel-like surface texture and gloss. The previous studies^7^ showed that the smoothest surface on resin composite restorations can be achieved by curing the material in direct contact with a smooth matrix surface (Mylar strip). To maintain surface finishing, further contouring and polishing are required.

Diamond burs having very fine particle size and white Arkansas stones were used to polish the resin composite restorations. As it has the ability to remove adjacent enamel, its use has been limited to initial contouring. Later on, most of the emphasis was placed on the application of fine grit abrasive discs to polish resin composites and also on the types of motion employed during their use.^8^ A rotary motion (circular), planar motion, and reciprocating motion can be used to polish the resin composites.

In a rotary motion (diamonds and cylindrical stones), the axis of rotation is parallel to the surface of the abrasive discs. The planar motion is a rotational movement. In the planar motion, the axis of rotation is perpendicular to the surface of abrasive discs. In reciprocating motion, a finishing strip is pulled back and forth over a surface.^9^

The results obtained by Fruits et al^9^ comparing different polishing motions on restorative materials showed that for all possible combinations, the planar motion showed the lowest average roughness values. In my study, a planar motion is used. Micro-filled and nanocomposites can be polished to produce an enamellike shine, while the conventional hybrid and packable composite resins used for posterior restorations can be polished so that it feels smooth as they do not require a high, enamel-like shine.^6^ According to Stoddard and Johnson,^10^ the effectiveness of finishing and polishing systems depends on the filler size and content, type of abrasives used, time spent with each abrasive, strokes, amount of pressure applied, the orientation of abrading surfaces, and the geometry (discs, cups, and stones) of abrasive instruments.

Turkun and Turkun^6^ compared the effects of Sof-Lex discs, Enhance and PoGo polishers on the surface of microhybrid resin composites. They reported that PoGo produced smooth surface as obtained by Mylar strips. These results were obtained because PoGo was used in combination with Enhance polishing system, as recommended by the manufacturers.

Enhance polishing system consists of a rubber-like flexible aluminum oxide particles and is a polymerized resin impregnated with an abrasive. This system may wear the resin matrix and only contour prominent surfaces, resulting in a higher surface roughness. PoGo system was applied after the use of Enhance polishing system that results in better surface smoothness.

Most investigators have concluded that flexible aluminum oxide discs are the best instruments for providing low roughness on composite surfaces.^11^ This study also showed smoother surfaces with Sof-Lex than PoGo polishers. These results may be due to the fact that initial finishing was not done with Enhance system.

In the present study, the smoothest surface was obtained with microhybrid resin composites using Mylar strip. These surfaces against Mylar strip were smoother than polished surfaces because the unpolished surfaces are composed of more polymer matrix than fillers. Turkun and Turkun,^6^ and Venturini et al^12^ demonstrated that aluminum oxide discs are capable of producing smooth surfaces, as they have the capacity to reduce fillers and matrix evenly.

This justifies that the multiple-step polishing systems were more effective in providing smoother surfaces for both microhybrid and nano-filled composites.^13^ The present results are similar to those of the study conducted by Watanabe et al,^14^ who showed that surface finishing with multiple-step polishing systems was superior to one-step polishing systems.

The single-step polishing system PoGo was used in this study without any surface pretreatment, as it reduces the time for contouring, finishing, and polishing the restorations, and it can be completed by using a single instrument that meets the clinical criteria for polishing a restoration in the minimal amount of time. This system presents higher surface roughness values as compared with the Sof-Lex discs.

In this study, stylus tip of a profilometer (Surftest SJ-201, Japan) was made to run on the surface of samples (groups I-IV). The tip of the stylus has a sensor that receives information from the test specimen and surface roughness value is digitally displayed. According to Quirynen et al,^15^ any increased surface roughness above 0.2 μm results in increase in plaque accumulation and increases the risk for periodontal inflammation and caries. Hooper et al^16^ studied the loss of hard tissue of about 0.5 μm with a contact profilometer. The composite surface cannot be fully characterized by the use of only surface roughness measurements as a parameter and conclusions cannot be drawn exclusively on the roughness average results.

## CLINICAL RELEVANCE

The clinical relevance was to check the surface roughness with the best polishing system on four newer composite resins.

## CONCLUSION

Mylar strip produced the smoothest surface of all the finishing and polishing systems. All finishing and polishing procedures decreased the smoothness obtained with matrix strips and resulted in Ra values above the threshold value of 0.3 μm.

Sof-Lex produces a smoother surface than PoGo finishing and polishing system. The effect of finishing and polishing systems on the surface of composites depends upon the material. Clearfil APX Esthetics showed significantly higher surface roughness than other nano-filled composite resins when PoGo was used as a polishing system. Synergy D6 showed significantly higher surface roughness when Sof-Lex was used as a polishing system.

Ceram X showed significantly higher surface roughness when a Mylar strip was used as a polishing system. The mean surface roughness was highest for Clearfil APX Esthetics (1.21 ± 0.19), followed by Synergy D6 (1.02 ± 0.21), Filtek Z 350 XT (0.79 ± 0.05), and Ceram X Mono (0.64 ± 0.05). This difference found was statistically significant (p < 0.001).

From the results, it was clear that the smoothest surface was obtained against a Mylar matrix. Statistical analysis shows that composite resins showed less surface roughness with Sof-Lex as compared with Pogo. Hence, within the limitations of this study, as Sof-Lex gives better results, it is recommended that it should be preferred as a polishing material.
